# Quality adjusted life years based on health and consumption: A summary wellbeing measure for cross‐sectoral economic evaluation

**DOI:** 10.1002/hec.4177

**Published:** 2020-10-22

**Authors:** Richard Cookson, Ieva Skarda, Owen Cotton‐Barratt, Matthew Adler, Miqdad Asaria, Toby Ord

**Affiliations:** ^1^ University of York York UK; ^2^ University of Oxford Oxford UK; ^3^ Duke University Durham North Carolina USA; ^4^ London School of Economics London UK

**Keywords:** cost effective, health, QALY, quality of life, wellbeing

## Abstract

We introduce a summary wellbeing measure for economic evaluation of cross‐sectoral public policies with impacts on health and living standards. We show how to calculate period‐specific and lifetime wellbeing using quality‐adjusted life years based on widely available data on health‐related quality of life and consumption and normative assumptions about three parameters—minimal consumption, standard consumption, and the elasticity of the marginal value of consumption. We also illustrate how these three parameters can be tailored to the decision‐making context and varied in sensitivity analysis to provide information about the implications of alternative value judgments. As well as providing a general measure for cost‐effectiveness analysis and cost‐benefit analysis in terms of wellbeing, this approach also facilitates distributional analysis in terms of how many good years different population subgroups can expect to live under different policy scenarios.

the UK governmental body NICE (the National Institute for Health and Care Excellence)…has performed a signal service. It has shown to the world that the wellbeing approach can become an acceptable basis for public policy.Lord O'Donnell, head of the UK civil service, 2005–2011 (O'Donnell, Deaton, Durand, Halpern, & Layard, 2014)

## 1 INTRODUCTION

Many public policies have important long‐run impacts on both health and living standards—including policies on social protection, education, employment and crime as well as health care and public health. In the health literature, policies of this kind are sometimes referred to as “cross‐sectoral” policies, because they have both health and non‐health effects and because they often have costs that fall on both health and non‐health budgets. We propose a summary outcome measure for the economic evaluation of cross‐sectoral public policies in terms of wellbeing—including cost‐effectiveness analysis, benefit‐cost analysis, and distributional analysis. This measure is proposed as an alternative (or complement) to standard benefit‐cost analysis and distributional analysis of cross‐sectoral policies in terms of money, and is not primarily intended as an alternative to standard cost‐effectiveness analysis of health care policies in terms of health.

Our proposed measure is a broader version of the quality‐adjusted life year (QALY) measure of health used in health economics (Cookson & Culyer, 2010). Instead of measuring years of healthy life—the health QALY—we propose to measure years of good life—the wellbeing QALY.^1^ There are many ways of creating a wellbeing QALY. One general approach is to adopt a single over‐arching “gold standard” questionnaire measure of wellbeing, such as life‐satisfaction (Frijters & Krekel, in press) or a multi‐dimensional quality of life instrument (Al‐Janabi, N Flynn, & Coast, 2012; Mukuria et al., 2018). Another is to create a “mash‐up index” of wellbeing which combines information from different sources on diverse health and non‐health outcomes using a wellbeing function or weighting system which precisely specifies how the combination is done. The weights can be set using a single source of data, such as a general population valuation exercise to set weights on different dimensions of health‐related and non‐health‐related quality of life (Mulhern et al., 2019). We adopt an even more thoroughgoing “mash‐up” approach, which uses multiple sources of data to parameterize the wellbeing function as well as multiple sources of data to measure the different dimensions of wellbeing, and which also takes care to ensure theoretical coherence by investigating the properties and implications of our wellbeing function and its theoretical basis in the “equivalence principle” of wellbeing valuation (Deaton & Muellbauer, 1980; Fleurbaey & Blanchet, 2013; Fleurbaey; Maniquet, 2011). Our specific proposal is to start with the conventional health QALY, which adjusts length of life for health‐related quality of life (HRQoL), and then make an additional adjustment for living standards, or income, or consumption.^2^

Our distinctive contribution is to construct a wellbeing measure that:

1. takes into account consumption as well as health
2. aligns with the widely used health QALY outcome measure from health economics
3. has theoretical foundations in contemporary welfare economic theory
4. respects standard theory and evidence about the diminishing marginal value of consumption, unlike standard cost‐benefit analysis without distributional weights
5. can be calculated based on readily available data on income and health

Constructing a wellbeing QALY metric using data on consumption and health is useful for two reasons. First, policy makers are often interested in effects on both consumption and health, and policy makers outside the health sector are often particularly interested in effects on consumption. Second, estimates of effects on consumption and health are often more readily available from trials and simulation modeling studies than estimates of effects on life satisfaction or multi‐dimensional quality of life.

Our wellbeing QALY measure is consistent with the “equivalence approach” to constructing wellbeing measures. The theoretical foundations of this approach have been extensively investigated in the welfare economics literature.^3^ The dimensions of health and consumption are combined in a way that aligns with welfare economic theory and also allows potential future extensions to incorporate other wellbeing dimensions (e.g., see Canning, 2013).

In conventional cost‐benefit analysis, non‐monetary outcomes are given a monetary value based on how much people are willing to pay for them, and then added up. This has two major limitations.

1. First, it makes no allowance for variation between individuals in the conversion rate from money to wellbeing—that is, the marginal value of consumption. Two particularly important sources of variation are that (1) an extra dollar does more to improve the wellbeing of a poor person than a rich person, and (2) an extra dollar is no use to anyone after their death
2. Second, it provides no information about the social distribution of costs and benefits and impacts on inequality

Although these limitations can in theory be addressed by applying “distributional weights” to monetary costs and benefits (Adler, 2016; HM Treasury, 2018), in practice this is rarely done. Our wellbeing QALY measure addresses these issues by taking into account standard assumptions about the diminishing marginal value of consumption.

The most obvious application of our approach is to public health policies which are primarily designed to improve health but also have important effects on income and consumption, such as “health taxes” on tobacco, alcohol and sugar. Such policies can be evaluated by combining epidemiological models of long‐term morbidity and mortality with economic models of supply and demand for the product under consideration. Models of this kind have been used in the United Kingdom, for example, to evaluate alcohol minimum pricing proposals for differential impacts by age, gender, alcohol risk group and income group. Once a combined epidemiological‐economic model of this kind has been built, one can then take the outputs—that is, effects on consumption, health and mortality by social subgroups—and convert them into wellbeing QALYs.

Our approach can also be applied to social policies which are not primarily designed to improve health—such as policies on education, social protection and the environment. Social policies often have important long‐run impacts on people's consumption, morbidity and mortality, even if those impacts are not the primary policy objectives. Social policies are currently evaluated using conventional cost‐benefit analysis, which translates all outcomes into monetary values. Wellbeing QALYs can be used to complement conventional cost‐benefit analysis by translating monetary costs and benefits into years of good life lost and gained. This is particularly useful when information is available about differences in costs and benefits by population subgroup, which is increasingly the case as sophisticated policy simulation models become more widely used to inform public health and social policy.

In addition, if in a particular policy application, the decision maker considers that there are important and potentially quantifiable outcomes not adequately captured by consumption, morbidity and mortality, then our approach can be extended in two ways. First, by adjusting quality of life directly for other outcomes—for example unemployment, crime, low life satisfaction, loneliness and so on. Second, by adjusting quality of life indirectly for other outcomes, by using a monetary value based on people's willingness to pay for improved outcomes and treating this as non‐market consumption that is then converted into years of good life gained.

In Section 2 we introduce the theory behind constructing the wellbeing QALY metric and discuss our key assumptions. In Section 3, we use examples to construct period‐specific wellbeing QALYs and distributions of lifetime wellbeing QALYs, as well as explore the key implications of using wellbeing QALY in economic evaluation. Section 4 discusses and concludes.

## 2 THEORETICAL FRAMEWORK

### 2.1 Period‐specific versus lifetime wellbeing

We can distinguish ‘period‐specific wellbeing’ from ‘lifetime wellbeing’.

We propose measuring period‐specific wellbeing $w_{i,t}$ as the time spent alive during that period, adjusted for overall quality of life (OQoL) during that period. It can be represented by a function $w_{i,t}\left(..\right)$ of both consumption and HRQoL during that period, that is:

(1) $$w_{i,t}=w_{i,t}\left(c_{i,t},h_{i,t}\right)$$

where *w*_*i,t*_ is the wellbeing or OQoL of individual i in period t; $c_{i,\;t}$ is the consumption of individual i in period t; $h_{i,t}$ is the HRQoL of individual i in period t; $w_{i,t}\left(c_{i,\;t},\;h_{i,t}\right)$ is monotonically increasing in both $c_{i,\;t}$ and $h_{i,t}$.

In theory the function $w_{i,t}\left(..\right)$ may vary by individual iand time period t. However, in practice a common population‐average wellbeing function will normally be used, just as a common population‐average health function is used to create a health index. As well as being convenient, there are also ethical arguments for this, to do with seeking to reflect general population average values (Hausman, 2010).

The assumption of additive separability of wellbeing over time is standard practice in economics and agrees with theories of lifetime wellbeing in the philosophical literature (Broome, 2004). Hence, we propose measuring lifetime wellbeing as the sum total of period‐specific wellbeing over the individual's lifetime:

(2) $$W_{i}=\overset{T_{i}}{\underset{t}{\sum }}w_{i,t}=\overset{T_{i}}{\underset{t}{\sum }}w_{i,t}\left(c_{i,t},h_{i,t}\right)\;$$

where $W_{i}$ is the lifetime wellbeing of individual i; $T_{i}$ is the time period that individual i dies; the other variables are as defined for Equation (1).

### 2.2 Interpreting one and zero

We propose to anchor the scale of period‐specific wellbeing in a way that (1) allows a QALY count of 1 to be interpreted as ‘a year of good life’ and (2) allows the use of readily available data on HRQoL. As in the standard QALY literature, we distinguish between the duration‐independent quality of life score and the period‐specific QALY count. We interpret a duration‐independent OQoL score of 1 as representing a state of wellbeing comprising both full health and a good standard of living (‘standard consumption’). The period‐specific QALY count for a period of time in that wellbeing state is then the duration of the period in years multiplied by the OQoL score (see discussion on this in section 2.5).

The standard consumption level can be tailored to the decision‐making context and the value judgments of the relevant decision makers in the same way as a poverty line or a value of statistical life. For national analyses, for example, it might be appropriate to set this at the national average level of consumption, whereas for global comparisons it might be useful to set standard consumption equal to the living standards of the average person in a modern high‐income country—a prosperous living standard in global historical terms, well above the global absolute poverty line though well below the highest attainable levels of affluence.

A duration‐independent OQoL score of 0 is assigned to a wellbeing state comprising the standard consumption level but in a severe state of ill‐health that is considered as bad as being dead. The same score of 0 is also assigned to a wellbeing state comprising full health with extremely poor living conditions considered as bad as being dead. We define “minimal consumption” as the extremely low level of consumption that represents these extremely poor living conditions—a concept of material and social deprivation potentially even more extreme than the subsistence level of consumption. To help distinguish this concept of extreme deprivation from standard concepts of poverty and subsistence, we sometimes use the phrase ‘minimal consumption for a life worth living’. Within our valuation system, various “in‐between” states can also be assigned a value of 0, for example, involving severe ill health (below full health) and low consumption (above the minimal level) which taken together are as bad as being dead.

The scale need not be bounded at either 0 and 1, allowing for the possibility of health and consumption states that are worse than being dead and levels of affluence that are better than a good standard of living. More specifically, as discussed in more detail later in the section on functional form and parameterization, states can be assigned ‘negative’ value (i.e., valued as ‘worse than being dead) either when HRQoL is extremely bad (a health state worse than being dead) or consumption is extremely low (a consumption state worse than being dead) or a combined state of severe illness and severe impoverishment that is overall considered worse than being dead. However, states ‘greater than 1’ can only arise when consumption is above the standard level. These assumptions would be a value judgment for the relevant decision‐making organization and could be varied in sensitivity analysis.

The above allows the wellbeing scale to be aligned with the zero point normally used in standard health QALY valuation exercises—that is, a state considered as bad as being dead—and facilitates the use of existing data on HRQoL to construct the wellbeing QALY (Devlin & Brooks, 2017). In principle, however, the zero point for both health and wellbeing QALY valuation exercises could instead be defined using states of health or wellbeing that are similar to being dead but not necessarily permanent, such as prolonged unconsciousness or a period of life barely worth living. To distinguish health QALYs and wellbeing QALYs, we will from now on refer to the health QALY as the health‐adjusted life year (HALY).

So far, our measure is similar to the HALY – except that we have re‐interpreted period‐specific quality of life as OQoL rather than HRQoL, and have potentially allowed scores ‘greater than one’ (in addition to ‘below zero’, which is already the case with the HALY).

### 2.3 Functional form and parameterization

To quantify wellbeing, we have to specify and parameterize the period‐specific wellbeing function $w_{i,t}\left(..\right)$. There are various possibilities for functional form (Hammitt, 2013).

Our proposal is the following simple additive wellbeing function:

(3) $$w_{i,t}=h_{i,t}+u\left(c_{i,t}\right)-1$$

where $u\left(c_{i,t}\right)$ is defined as:

(4) $$u\left(c_{i,t}\right)=A-B\times {\left(c_{i,t}\right)}^{1-\eta }$$

with η>1 (‘eta’) set to represent the elasticity of the marginal value of consumption, and *A* and *B* set as normalization constants to ensure that the utility scale is appropriately anchored so that 1 is a state of ‘good life’ and 0 is a state ‘as bad as being dead’.

More specifically, we assume $A\;=\;c_{min}^{\left(1\;–\;\eta \right)}\;/\;\left(c_{min}^{\left(1\;–\;\eta \right)}\;-\;c_{std}^{\left(1\;–\;\eta \right)}\right)$ and $B\;=\;1\;/\;\left(c_{min}^{\left(1\;–\;\eta \right)}\;-\;c_{std}^{\left(1\;–\;\eta \right)}\right)$, where:

1. $c_{std}$ is standard consumption for a good standard of living, set at a level that the relevant social decision makers consider to represent a good living standard;
2. $c_{min}$ is minimal consumption for a life worth living, set at a level that the relevant social decision makers consider to represent a living standard as bad as being dead.^4^

In Equation (4), the normalization constant, −1, anchors wellbeing to 1 when health quality, h, is 1 and consumption is standard consumption; to 0 when health quality is 1 and consumption is minimal consumption; and to 0 when health quality is 0 and consumption is standard consumption.

The higher the “eta” parameter, the more rapidly diminishing returns set in as consumption increases. The theoretical literature on isoelastic functions supports the possibility that *η* ≤ 1, in which case the wellbeing function is not bounded from above. However, the empirical literature supports values of *η* of at least 1 (Layard, Mayraz, & Nickell, 2008).

The simple additive wellbeing function assumes the marginal benefit of consumption does not depend on ill health (Bleichrodt & Quiggin, 1999; Hammitt, 2013; Smith & Keeney, 2005). An alternative view might be that the marginal benefit of consumption increases with ill health. For example, someone unable to walk might gain considerable benefit from consuming mobility equipment and a variety of transport, communication and personal care services—at least some of which might not be picked up in standard measures of health benefit. Yet another view might be that the marginal benefit of consumption decreases with ill health. For example, additional consumption of material goods and services may bring limited benefit to someone who is severely depressed and no longer able to enjoy material consumption. In Appendix S1B, we explore a more complex functional form for the period‐specific wellbeing function, which allows for simple interactions between health quality and consumption. A further issue which we do not explore in Appendix S1B is the possibility of more complicated interactions between health quality and consumption which differ for different dimensions of health—for example, the possibility that mental and physical dimensions of health interact with consumption in different ways to produce wellbeing.

These are important challenges for future empirical work, but for the time being we do not propose the use of wellbeing functions with consumption‐health interactions because of the lack of empirical evidence about how far consumption and different dimensions of health are substitutes or complements, as well as the added complexity this brings to practical application of a wellbeing measure. There is not much empirical evidence about this issue (Evans & Viscusi, 1991; Rey & Rochet, 2004) and the findings are mixed with some studies finding that health and consumption are complements (Viscusi & Evans, 1990) and others finding they are substitutes (Tengstam, 2014).

### 2.4 Measuring consumption and health quality

The level of consumption $c_{i,t}$ in Equation (1) can be expressed in real financial resources—such as dollars in a given time period. Since we normalize the wellbeing QALY to units of one year, it will generally be convenient to measure consumption in annual units (e.g., converting weekly or monthly consumption data to the corresponding annualized figures); though we discuss the issue of time periods shorter than one year in section 2.5.

Consumption can be measured in different ways, depending on the purpose of the analysis and the availability of data. Consumption is generally defined as the market value of the goods and services used in a given time period. A broad measure of consumption, for example, would include not only the market value of all purchased goods and services but also the imputed market value of goods and services provided or subsidized by the state—such as health, education and local public amenities—and by the family and others—such as housing, cooking and informal care. Consumption can differ from income, due to savings and bequests. In practice, however, income is often a useful proxy for consumption and income can enhance wellbeing by providing financial security, which can be thought of as a form of beneficial consumption.

Health quality in time period t, that is, $h_{i,t}\;$in Equation (1) is measured using the standard measure of HRQoL over the relevant time period t from the HALY literature. This means it can be represented by a function $h_{i,t}\left(H_{i,t}\right)$, where $H_{i,t}$ is a multi‐dimensional vector of the HRQoL attributes of individual i in time period t; and $h_{i,t}$ is a scalar measured on the standard scale of the HRQoL over the relevant time period, anchored at 1 representing full health, and at 0 representing a state equivalent to being dead (Devlin & Brooks, 2017; Devlin, Shah, Feng, Mulhern, & van Hout, 2018; Dolan, 1997; Van Hout et al., 2012). We use the phrase “health quality” to emphasize that $h_{i,t}$ is not a value‐free physical quantity of health but a value‐laden index of HRQoL that requires value judgments both in selecting and describing the relevant dimensions of health and in combining measurements of the different health dimensions to generate an overall index score.

It may be argued that incorporating consumption and HRQoL as part of an additive wellbeing measure can create a risk of double counting, in two ways. First, individuals may use some of their income on maintaining or improving their HRQoL through out‐of‐pocket purchase of health care or equipment or social services that improve one or more dimensions of health quality (such as mobility, self‐care, pain, usual activities, anxiety). Because of this, arguably one may want to define consumption as net of health‐related personal expenditure. However, it is not clear how important this would be in practice—especially in high‐income countries with well‐developed insurance systems. To make a large difference the amount of out of pocket expenditure would have to be a substantial proportion of total income. Second, when responding to standard HRQoL valuation exercises people may implicitly assume that improved health quality will also yield increased income and consumption, and this may influence their valuations of some health states. If so, arguably we should make an adjustment to health quality scores before using them for wellbeing QALY purposes, to strip out any assumptions that respondents may be making about consequent increases in income caused by increases in health. This may be a fruitful avenue for future research, to investigate what difference this makes and how far HRQoL valuation responses in different countries are influenced by assumptions about the income effects of improved health quality.

### 2.5 Valuing wellbeing during sub‐periods shorter than 1 year

To be consistent with the literature on HALYs, we have suggested normalizing the wellbeing measure in units of one year and labeling this a year of good life or a wellbeing QALY. The wellbeing QALY is analogous to the HALY but is a broader concept that it is adjusted for OQoL rather than just HRQoL. In our proposed version of the wellbeing QALY, this involves adjusting for consumption as well as HRQoL, while in other versions this can involve adjusting for life‐satisfaction or for a multi‐dimensional questionnaire measure of OQoL.

Normalizing in units of one year is compatible with measuring wellbeing and its components during time periods shorter than one year. In the standard HALY literature, it is common to collect data on HRQoL covering sub‐periods of time shorter than 1 year (e.g., at baseline and various follow‐ups). This data can then be aggregated to yield the total HALY count, applying methods to handle the discrete changes over time (see Manca, Hawkins, & Sculpher, 2005 for a discussion). We suggest estimating the HRQoL component of our wellbeing measure in Equation (3), that is, $h_{i,t}$ using these same standard HALY methods.

This makes it important to distinguish between the duration‐independent HRQoL and the duration dependent HALY count. The former is a duration‐independent value attached to a health state and the latter is a duration dependent count that is normalized in time period units of one year. The HALY count can be expressed as the duration‐weighted sum of the HRQoL scores experienced during all the relevant periods (or sub‐periods) of time—for example, a half year lived at a HRQoL of 1 generates a HALY count of 0.5 HALYs.

The same distinction applies to the wellbeing QALY—we can distinguish the duration‐independent OQoL score from the duration dependent QALY count. The distinction is also applicable in theory to the value of consumption, which can be measured in time periods shorter than one year (e.g., weekly or monthly consumption) though is also often measured using data on annual income as a proxy indicator. However, it would be unusual to measure consumption over time periods shorter than a day, and the concept of “instantaneous” consumption is somewhat problematic from a practical perspective. For example, imagine time periods were measured in minutes. Straight after consuming a hearty meal, your wellbeing is likely to depend more on consumption in the previous few minutes, and perhaps on anticipated consumption later in the day, than on consumption in the current minute—thus making it implausible to assume that the value of instantaneous consumption is additively separable over time periods shorter than a day. The assumption of additive separability is less problematic, however, when consumption is measured over longer time periods, such as months or years.^5^ We therefore suggest measuring consumption using the best available data—which in many cases may be data on income—and then converting to the corresponding annual consumption figure if this data is not already in annual units. Always working with annualized consumption figures means that the same normative parameters for minimal consumption, standard consumption and eta can be used, without having to re‐scale everything to a sub‐annual consumption scale.

## 3 ILLUSTRATIONS

In this section, we illustrate how to construct wellbeing QALYs in two simple examples, by specifying potential normative parameter values and exploring their implications. We start with a global example that might be relevant to decision making by an international organization, before turning to a national example in a UK decision‐making context. We use our global example to show how period‐specific wellbeing varies with consumption, to assess the implications for the consumption value of health, and to contrast our wellbeing valuation approach to the monetary valuation approach used in standard benefit‐cost analysis. We then use our UK example to demonstrate how the metric can be used for distributional analysis in terms of lifetime wellbeing.

### 3.1 Choice of normative parameters

We need to set three normative parameter values—minimal consumption for a life barely worth living, standard consumption for a good living standard, and the elasticity of the marginal value of consumption. In this section, we choose the parameters consistent with our global example, but a similar method would yield parameters consistent with our UK example.

First, we set the minimal consumption value, $c_{min}$. We start with the World Bank's current absolute global poverty line of $1.90 a day in 2011 prices (updating the previous line of $1.25 a day in 2005 prices), corresponding to $693.50 per year in 2011 prices (Ferreira et al., 2016). Since we normally think that healthy lives in absolute poverty are worth living, we set $c_{min}$ below this level. So in the example below we use a value of $c_{min}$ = $300 per year.

For $c_{std}$, we use a value of $30,000 in 2014 prices, based on the following calculation and using income as an indicator of consumption. In 2014, US median household annual income before taxes and benefits was $53,657, average household size was 2.6 and 23% of the population were children (aged 0 to 17) (DeNavas‐Walt & Proctor, 2015; Lofquist, Lugailia, O'Connell, & Feliz, 2012). We can thus think of the average household as comprising 2 adults and 0.6 of a child. To allow for household size and composition, the standard equivalence scale used in the United States for this kind of household is (adults + 0.5 × children)^0.7^ which yields a scale of 1.79 (“Equivalence Adjustment,” 2016). Dividing household income by 1.79 then gives us a figure of $29,951 for individual income, which we round up to $30,000.

We use this figure for convenience, as ideally one would want a figure after taxes and benefits and including the value of “in kind” benefits and services from the state and family. This figure is not an unreasonable starting point, however, insofar as the taxes paid to the state by the typical household can be assumed approximately equal in value to the cash and noncash benefits received from the state. It will nevertheless underestimate the broad concept of individual consumption that we might ideally wish to measure, since it excludes the value of informal household services such as cooking, cleaning, childcare and so on.

Finally, we set ‘eta’ equal to 1.26 based on a study of the association between subjective wellbeing and consumption by Layard et al. (2008), using four large cross‐sectional surveys and two panel surveys from multiple countries between 1972 and 2005.

In Appendix S1C, we investigate the implications of different assumptions about the above parameters.

### 3.2 Implications for the marginal value of consumption and health

**FIGURE 1:**
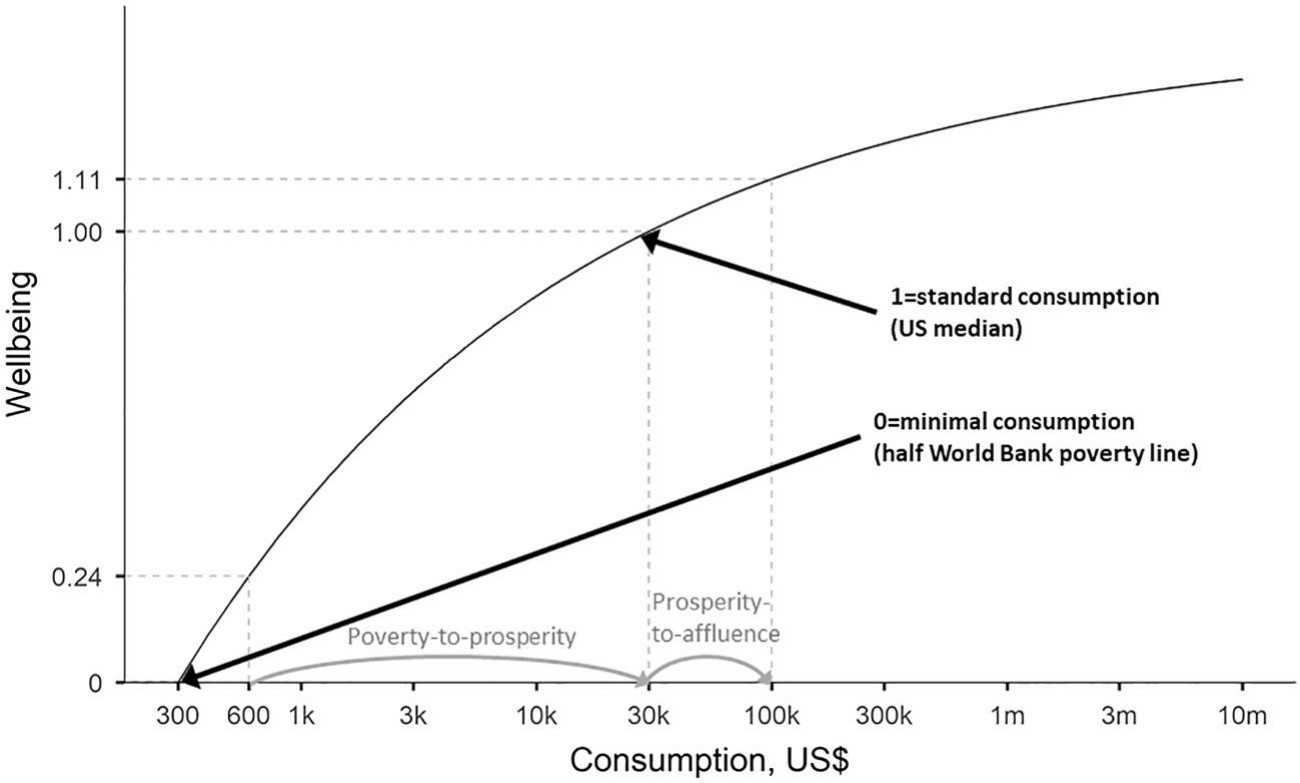
Wellbeing value of consumption in full health. Note: Consumption shown on a log scale. Wellbeing is depicted for a person in full health, assuming the following parameters: minimal consumption (for a life barely worth living) $c_{min}$ = $300, standard consumption (for a good living standard or “prosperity”) $c_{std}$ = $30,000 and elasticity of the marginal value of consumption η = 1.26. We also mark the “poverty” level of consumption at $c_{pov}$ = $600 and the “affluence” level of consumption at $c_{aff}$ = $100,000, and compare the improvements in living standard when moving from poverty to standard consumption (“poverty‐to‐prosperity”) and when moving from standard consumption to affluent consumption (“prosperity‐to‐affluence”)

Figure 1 shows what the resulting relationship between period‐specific wellbeing and consumption for someone in full health would look like, under these parameter assumptions.^6^ To help understand the implications of these parameter values, it is useful to consider two further normative consumption thresholds—poverty line consumption, $c_{pov}$, below which the individual is considered poor and “affluence line” consumption, $c_{aff}$, above which the individual is considered affluent (i.e., having a great standard of living as opposed to a merely good standard of living). Setting these thresholds allows us to compare the value of two benchmark improvements in living standards: (1) from poverty line consumption to standard consumption (“poverty‐to‐prosperity”) and (2) from standard consumption to affluent consumption (“prosperity‐to‐affluence”).

Minimal consumption for a life barely worth living is well below the poverty line level of consumption that a modern high‐income country government would consider acceptable for its poorest citizen. In practice, minimal consumption would be hard to sustain in a high‐income country as the market price of basic food and shelter is substantially more than $1 a day and our concept of consumption includes the imputed value of goods and services provided for free. So subsisting on minimal consumption would require avoiding offers of food and shelter from the state, family, friends or kind strangers and living like a lone wild animal: not just sleeping rough and foraging for food but also avoiding almost all social contact.

We can contrast the implications of our wellbeing metric with a conventional monetary approach. Table 1 compares two consumption gains that we refer to as “poverty‐to‐prosperity” and “prosperity‐to‐affluence”. By “poverty‐to‐prosperity” we mean increasing a person's annual consumption level from $600 (the World Bank absolute poverty line) to $30,000, a prosperous living standard set approximately equal to the average living standard in the USA. This scenario implies a gain of $29,400 in monetary terms and, under our assumptions, a gain of around 0.76 (=1.00−0.24) wellbeing QALYs (see Figure 1). By “prosperity‐to‐affluence” we mean increasing a person's annual consumption level from $30,000 (a prosperous living standard) to $100,000, equal to an affluent living standard in a high‐income country. This scenario implies a gain of $70,000 and around 0.11 wellbeing QALYs.

**TABLE 1:** Wellbeing QALYs versus conventional unweighted monetary valuation

Concept	Poverty‐to‐prosperity	Prosperity‐to‐affluence
Definition	Moving someone from $600 to $30,000 annual consumption	Moving someone from $30,000 to $100,000 annual consumption
Gain, unweighted US$	29,400	70,000
Relative valuation	0.42 (prosperity‐to‐affluence is more important than poverty‐to‐prosperity)	
Gain, wellbeing QALYs	0.76	0.11
Relative valuation	6.57 (poverty‐to‐affluence is more important than prosperity‐to‐affluence)	

*Note*: The “relative valuation” represents the gains from “poverty‐to‐prosperity” divided by the gains from “prosperity‐to‐affluence”, as defined above. The calculations are based on a person in full health, assuming minimal consumption (for a life barely worth living) $c_{min}$ = $300, standard consumption (for a good living standard or “prosperity”) $c_{std}$ = $30,000 and elasticity of the marginal value of consumptionη = 1.26.

According to conventional unweighted monetary valuation, a prosperity‐to‐affluence gain in consumption is more than twice (1/0.42 = 2.38) as valuable as a poverty‐to‐prosperity gain. So according to conventional cost‐benefit analysis, it is better to give $70,000 to an already prosperous person with a consumption of $30,000 rather than to give $29,400 to a person living in absolute poverty. However, in terms of wellbeing QALYs, the poverty‐to‐prosperity gain is worth almost seven times the prosperity‐to‐affluence gain—a diametrically opposed implication.

This example illustrates the scale of the difference our approach would make in practice to assessments of programs with differential benefits for different income groups. These differences would be masked by conventional cost‐benefit analysis without any distributional impact assessment.

We also show that QALY valuation will always give relatively more importance to poverty‐to‐prosperity than monetary valuation, no matter how poverty and affluence are defined and no matter what values are chosen for the parameters $c_{min},\;c_{std}$ and η—except for the polar extreme case where η is set equal to zero and there are no diminishing returns to consumption (see Appendix S1E).

We can also calculate the marginal rate of substitution (MRS) of consumption for health by dividing the marginal value of the latter by the marginal value of the former. The marginal value of health is simply 1, and the marginal value of consumption is $-B\;\left(1\;–\;\eta \right)c^{-\eta }$. So we get: $MRS\;=\;c^{\eta }/\;\left(B\times \left(\eta \;-1\right)\right)$.

**FIGURE 2:**
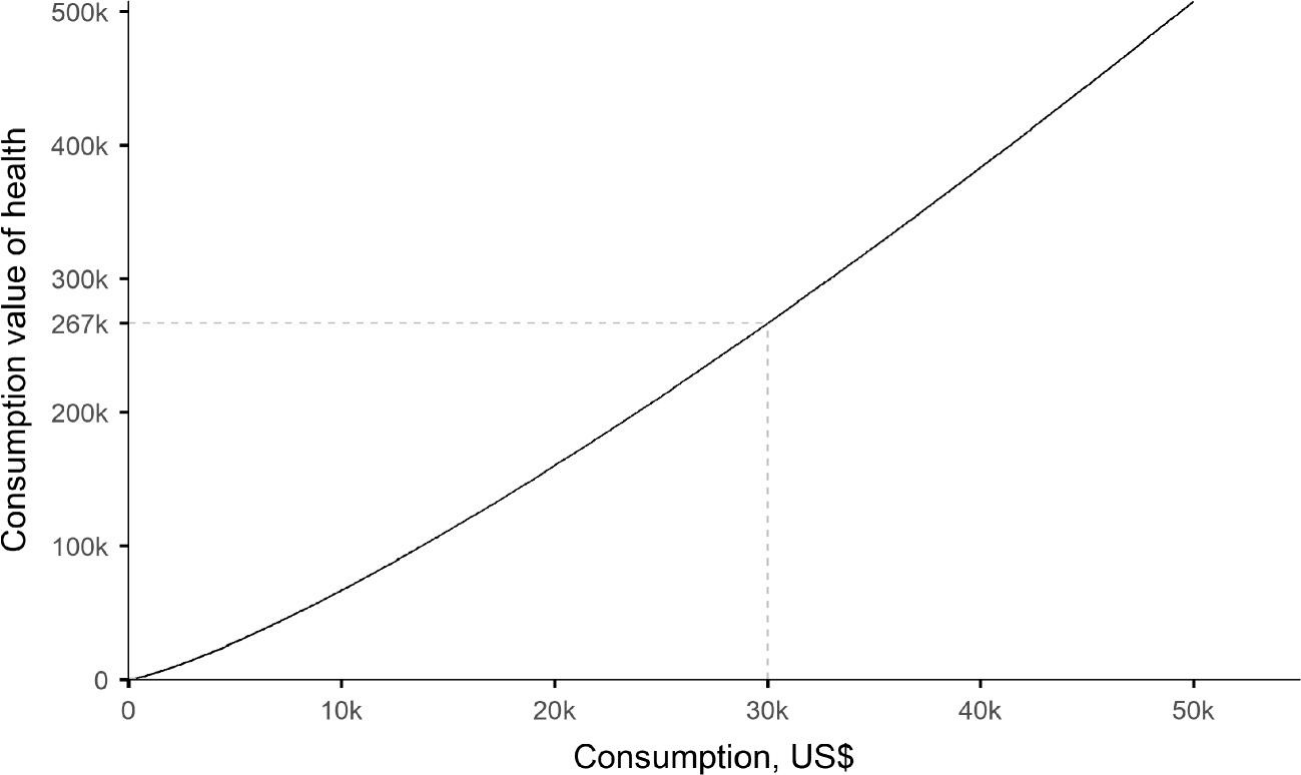
Implied marginal consumption value of a HALY. Note: The assumptions for the parameters of the wellbeing function are as follows: minimal consumption (for a life barely worth living) $c_{min}$ = $300, standard consumption (for a good living standard) $c_{std}$ = $30,000 and elasticity of the marginal value of consumption η = 1.26

This MRS represents the implied marginal consumption value of health, given different levels of consumption. Figure 2 shows implied values for our base case parameters, with the consumption value of health on the vertical axis. This shows that, according to our model, rich individuals should be willing to sacrifice substantially larger amounts of consumption than poor individuals to gain an additional year of healthy life. For example, an individual at the minimal level of consumption ($300 dollars a year) should be willing to sacrifice consumption for health at an exchange rate of $805 per HALY, whereas an individual at a standard level of consumption ($30,000 a year) should be willing to pay $267,000 per HALY. If we invert these figures to consider willingness to pay in health for gains in consumption, this shows that one dollar of additional consumption is worth substantially more to a poor individual than a rich individual.

### 3.3 Lifetime wellbeing distributions

Decision makers are often interested in distributional impacts as well as total costs and benefits. The wellbeing QALY allows us to provide a general measure of distributional impact on lifetime wellbeing, as well as domain‐specific measures of distributional impact on consumption, health and other specific outcomes.

Such analysis, however, requires the availability of individual level data on the two wellbeing dimensions—health quality and income—over the whole lifecourse. Simulation modeling can generate such datasets, by combining experimental and non‐experimental data. To illustrate this, we use the lifecourse microsimulation model LifeSim which provides detailed information about the distribution of long‐term policy outcomes between population groups or individuals, by simulating a wide range of life outcomes from birth to death of 100,000 British children born in year 2000–2001.^7^

We use simulated annual data on consumption and HRQoL to generate the period‐specific metric of wellbeing for each individual‐life year consistent with Equation (1) and the additive specification in Equations (3) and (4). We then aggregate the period‐specific wellbeing measures consistent with Equation (2), to generate a lifetime wellbeing measure for each member of the cohort.

In this application, we tailor the minimal consumption parameter to a UK context. The average UK household spends around £26.34 per person per week on food (excluding eating out and alcohol), which equals £1371 per year (National Statistics, 2018). We set the minimal consumption parameter slightly below this at £1000 per year, which we assume would be an intolerable level of material and social deprivation for most UK citizens, requiring sleeping rough for a year with no social interaction and a bare minimum of food for survival. We set the standard consumption parameter at £24,000 per year, as that is the mean consumption level predicted by LifeSim for the particular cohort. As before, we set the value of ηat 1.26.

**FIGURE 3:**
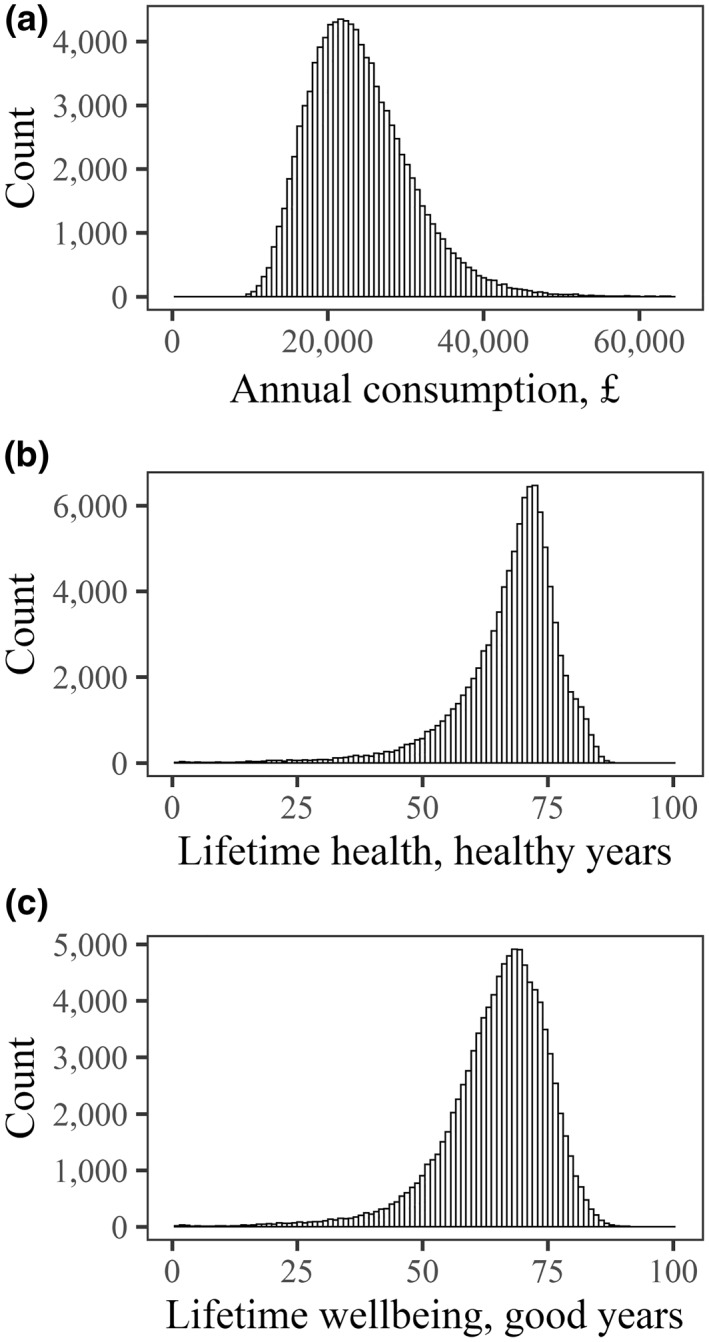
Distributions of annual consumption, lifetime health and lifetime wellbeing for a simulated British cohort. Panel A: Distribution of average annual consumption over lifetime. Panel B: Distribution of lifetime health. Panel A: Distribution of lifetime wellbeing. Note: The distributions summarize detailed life histories for 100,000 simulated individuals. The assumptions for the parameters of the wellbeing function are as follows: minimal consumption (for a life barely worth living) $c_{min}$ = £1,000, standard consumption (for a good living standard) $c_{std}$ = £24,000 and elasticity of the marginal value of consumption η = 1.26

Panels A and B of Figure 3 depict the distributions of annual consumption and lifetime health quality across the simulated cohort, and Panel C shows the resultant distribution of lifetime wellbeing quantified in wellbeing QALYs.

The equity impact of a policy on the distribution of lifetime wellbeing can then be assessed using various equity metrics, as discussed in Appendix S1F. For example, one can compute indices of inequality in experienced lifetime wellbeing between individuals, and differences in expected years of good life at birth between different population subgroups.

## 4 DISCUSSION

### 4.1 Summary

We have proposed a practical way of evaluating cross‐sectoral policies by combining data on consumption, HRQoL and mortality to measure years of good life or wellbeing QALYs. This general wellbeing measure could in principle be used in any type of economic evaluation, including cost‐effectiveness analysis (assuming a fixed government budget), cost‐benefit analysis (with all benefits and opportunity costs valued in terms of wellbeing QALYs), and distributional analysis.

Our approach requires estimates of policy effects on consumption, HRQoL and mortality by social group, and explicit specification of three new normative parameters:

1. elasticity of the marginal value of consumption, η
2. minimal consumption for a life barely worth living, $c_{min}$
3. standard consumption for a good living standard, $c_{std}$

These three parameters specify how much wellbeing is derived from any given level of consumption, for a person in full health. The first specifies the degree of curvature in the curvilinear relationship between consumption and wellbeing, the second specifies where it crosses the horizontal axis at zero wellbeing, and the third specifies the standard level of consumption in full health that is considered to represent a year of good life. Taken together, these parameters tell us how much change in wellbeing is derived from a one dollar change in consumption, and how this varies for people with different baseline levels of consumption.

Like other normative parameters in economic evaluation, such as the discount rate and the cost‐effectiveness threshold, these parameters are ultimately a matter for value judgment by the relevant social decision makers, after a due process of public deliberation. To help guide this process of deliberation, empirical benchmarks can be found for all three parameters and the implications of different parameter values can be explored in sensitivity analysis. For example, benchmarks for η could be found using data on life satisfaction and trade‐offs between consumption and health, benchmarks for $c_{min}\;$and $c_{std}$ using data on willingness to pay for full health and to avoid extremely bad health states, and other more ingenious approaches might be found. The search for empirical benchmarks suitable for use in different decision‐making contexts thus opens up an interesting and wide‐ranging agenda for future research in this area.

### 4.2 Strengths and limitations

The attractive features of our approach are:

1. it builds on and aligns with the widely used health QALY (HALY) outcome measure from health economics
2. it has theoretical foundations in contemporary welfare economic theory
3. it uses a simple, intuitive metric—years of good life
4. it can be applied to any decision with impacts on health or consumption or both
5. it requires only widely available data on income, health and mortality
6. it encourages social decision makers and stakeholders to specify their value judgments and explore the implications of alternative value judgments
7. it can be used to produce distributional breakdowns in terms of lifetime wellbeing
8. it allows analysis of percentage changes and equity‐efficiency trade‐offs

The main disadvantages compared with standard cost‐benefit analysis without distributional weights are

1. it has more demanding data requirements for estimates of individual‐ or group‐level costs and benefits in terms of consumption and health (also known as “distributional analysis”), as well as average costs and benefits
2. it has more demanding normative requirements for explicit social value judgment by social decision makers (i.e., the flip side of advantage 6).

Although more demanding in terms of data, distributional analysis is becoming ever more feasible in the age of “big data” (Layard, Clark, Cornaglia, Powdthavee, & Vernoit, 2014; Skarda; Asaria, & Cookson, 2020; M. Wolfson & Rowe, 2014; M. C. Wolfson, 1995; M. C. Wolfson & Rowe, 2013). Furthermore, a thoroughgoing application of conventional cost‐benefit analysis would also require distributional analysis, since willingness to pay depends on baseline consumption and health. It is just that in practice distributional modeling is rarely if ever done—instead, the analysis relies upon population average estimates.

Whether demanding requirements for decision makers to make strong and explicit social value judgments is an advantage or a disadvantage depends on one's underlying political philosophy. One view is that this is an advantage, since it facilitates transparency and public accountability. An opposing view is that this is a disadvantage, because it may fail to respect individual preferences. The wellbeing QALY approach could be adjusted to reflect individual preferences as closely as possible, by adjusting the normative parameters in line with empirical evidence. A difficulty here—which afflicts all approaches that attempt to respect individual preferences—is that empirical evidence about individual preferences is inconsistent, because behavior can be powerfully influenced by apparently irrelevant factors such as “priming” and “framing” effects (Kahneman, 2011; Sugden, 2008). However, it may be possible to find acceptable ways of “laundering” the actual preferences that motivate behavior to discover a well‐informed and logically coherent set of underlying preferences suitable for economic evaluation (Adler, 2012; Goodin, 1995).

We have proposed the wellbeing QALY based on consumption and health for use in economic evaluation of cross‐sectoral public policies with important impacts on both income and health, where there is a clear need to compare impacts on both dimensions of wellbeing. We do not necessarily recommend its use for economic evaluation of healthcare technologies and programs—especially in cases where policy makers are primarily concerned about impacts upon health and hence the standard HALY is sufficient—though it might find useful application in cases where policy makers are concerned about impacts on financial protection as well as health. And we certainly do not recommend using it to guide individual‐level “bedside rationing” decisions about which patient to treat, since in that context the adjustment for consumption could be misused to justify pro‐rich discrimination in the provision of effective life‐extending treatments. For example, under the illustrative assumptions for global comparisons set out above, extending the life of a rich individual with $100,000 consumption would yield 0.11 more wellbeing QALYs per year than extending the life of someone with the same health quality but on average income, and extending the life of a super‐rich individual with $10m annual consumption would yield 0.3 more wellbeing QALYs per year. Similar issues about the potential for discrimination afflict the HALY, of course, since in theory HALYs can be used to justify discriminating against disabled or frail or co‐morbid individuals with low HRQoL in the provision of effective life‐extending treatments. Like all tools, the HALY and the wellbeing QALY can be misused: they should be used in the right way for the right jobs.

### 4.3 Comparison with other approaches

The leading alternative to the “wellbeing QALY” approach is the “equivalent income” approach (Fleurbaey, Luchini, Muller, & Schokkaert, 2013). This approach retains money as the metric of value but uses a system of distributional weights to adjust the raw willingness to pay amounts, and supplements this with survey data on how much people are willing to pay for full health. One advantage of this approach over wellbeing QALYs is that it only requires one explicit normative parameter from the social decision maker—aversion to inequality in equivalent income—and otherwise in principle respects individual preferences. A disadvantage is that distributionally weighted income figures are somewhat unintuitive for policy makers, whereas experience in the health field has shown that decision makers are capable of understanding and using the QALY concept despite initial qualms.

There are various other ways of constructing “wellbeing QALY” type measures. O'Donnell et al. (2014) have proposed measuring period‐specific wellbeing directly using data on life satisfaction. One way of doing this is to define the basic unit as a one‐point improvement in life satisfaction for one person for one year, which some authors refer to as a WELLBY (Frijters & Krekel, in press). If life satisfaction is measured on a ten‐point scale, and if 2 is considered to represent a life barely worth living while 10 is a fully satisfactory life, then one WELLBY is approximately one eighth of a wellbeing QALY. One advantage of treating life satisfaction as a direct “gold standard” measure of wellbeing is that distributional information about consumption is not necessarily required. A disadvantage, however, is that interpretation of life satisfaction as a ratio scale variable is an ad hoc assumption that so far has only been subjected to limited psychometric testing—in contrast to the large literature on developing and testing ratio scale measures of health quality (Brazier, Ratcliffe; Saloman, & Tsuchiya, 2007). Another issue is that data on life satisfaction impacts of policies are less frequently collected than data on health and consumption outcomes, and the research community so far has limited experience using data on life satisfaction directly to measure policy outcomes—so potential biases around issues such as expectations, adaptation and set points have not been fully explored in the context of policy evaluation (Di Tella; MacCulloch, 2006; Fujita & Diener, 2005; Lucas, 2007). However, even if life satisfaction outcomes are not directly measured in policy evaluation, data on life satisfaction can still be used indirectly to set a wellbeing value on the outcomes that are measured – as we have done in the present study to parameterize the elasticity of the marginal value of consumption.

Another way of creating a wellbeing QALY would be to use a multi‐dimensional quality of life questionnaire (Al‐Janabi et al., 2012; Mukuria et al., 2018), and find a way of converting multi‐item survey responses into a ratio scale with suitable anchoring at 0 and 1. Multi‐dimensional quality of life questionnaires are not (yet) in widespread use in policy evaluation. In principle, however, this approach could be used indirectly to set a wellbeing value on other outcomes that are measured.

How far wellbeing QALYs constructed in these three different ways are comparable is then an important issue for future research. Table 2 summarizes the key features of these various approaches.

**TABLE 2:** Key features of the four leading alternatives to standard cost‐benefit analysis

Approach	Main data sources	Main explicit normative parameters	Metric
Health and consumption QALY	Consumption, health	Elasticity of marginal utility of consumption, standard consumption, and minimal consumption	Years of good life
Life satisfaction QALY	Life satisfaction	None—though embodies normative assumptions in treating ordinal data as a ratio scale	Years of good life
Multi‐dimensional questionnaire QALY	Multi‐dimensional quality of life questionnaire	None—though embodies normative assumptions in collapsing multi‐item survey responses to a ratio scale	Years of good life
Equivalent income	Willingness to pay, including willingness to pay for full health	Aversion to income inequality	Distributionally weighted income

Abbreviation: QALY, quality‐adjusted life year.

### 4.4 Implications for research

Our wellbeing measure can be used as a practical summary measure in applied research on cross‐sectoral economic evaluation, whenever estimates are available of individual‐ or group‐level policy impacts on both consumption and health.

All approaches to measuring long‐term wellbeing impacts require models of the long‐term effects of policies on different dimensions of wellbeing for different types of individual. A key next stage for research will therefore be to develop microsimulation models of individual wellbeing over the lifecourse and use them to apply these novel wellbeing metrics in practice and assess their added value in providing decision makers with useful new information (Skarda, Asaria, & Cookson, 2020).

In addition, as for particular policy applications there may be important and potentially quantifiable outcomes that are not adequately captured by long‐term effects on consumption, morbidity and mortality, it would be of interest to further extend our approach by incorporating further dimensions of wellbeing besides consumption and health. The simplest approach would be to include an additive quality of life score for various indicator variables representing “good” or “bad” life outcomes, whereby there is a wellbeing increment or decrement for each event—for example, a wellbeing loss for loneliness, unemployment, homelessness and so on. There are risks of double counting, however, if the modeling is done in a naive way that does not allow for dynamic causal interactions between outcomes in different time periods (e.g., mental illness and unemployment) and simply counts both outcomes twice. There may also be static value interactions within the wellbeing function—for example, the wellbeing loss of having mental illness and unemployment at the same time may not simply be the sum of the wellbeing loss of each outcome in isolation. These are matters for future research, requiring careful scientific modeling and careful consideration of interactions between different arguments in the wellbeing function.

Useful steps for future research in refining our wellbeing QALY approach would also include general population stated preference valuation exercises to estimate the three parameters in our wellbeing function. Finally, it would be useful to compare how our wellbeing metric compares with other methods of measuring wellbeing discussed, including equivalent income, the life‐satisfaction approach and various multi‐dimensional quality of life questionnaire approaches.

## CONFLICT OF INTEREST

Dr. Cookson reports grants from NIHR, grants from Wellcome Trust, during the conduct of the study. Dr. Skarda reports grants from NIHR, grants from Wellcome Trust, during the conduct of the study. The other authors have no conflict of interest to declare.

## AUTHOR CONTRIBUTIONS

The original idea arose from conversations between Toby Ord, Owen Cotton‐Barratt and Richard Cookson and was further developed following conversations with Matthew Adler. Richard Cookson drafted the original working paper version and co‐drafted the revised journal article version. Owen Cotton‐Barratt and Matthew Adler formulated the wellbeing equations and Miqdad Asaria created the working paper graphs. Ieva Skarda re‐structured the working paper into journal article format and added substantial new analysis, graphs and equations. All authors contributed to study design, interpretation and writing up and have approved the final manuscript.

## Supporting information

Supplementary Material

## ENDNOTES

^1^
We have no strong views on terminology, other than seeking to avoid an acronym with off‐putting connotations such as WALY (wellbeing‐adjusted life year). Our preference would be to switch to using health‐adjusted life year (HALY) for the narrower concept of a healthy year of life, and to reserve quality‐adjusted life year (QALY) for the broader concept of a good year of life. However, we realize that use of the acronym QALY for the narrower health concept is deeply ingrained in the health literature.

^2^
Consumption is the market value of the goods and services used by an individual in a time period. Consumption can differ from income since it can be provided by in‐kind transfers, or financed by borrowing or selling assets, and income can be given away or saved. Income is easier to measure than consumption though often more volatile.

^3^
Please see Appendix S1A for a discussion how our wellbeing QALY is consistent with the “equivalence approach” of wellbeing valuation.

^4^
This does not necessarily mean it would be better for the person to die, since death is permanent whereas we are here only valuing a temporary period of time alive. Rather than thinking of zero wellbeing as being “as bad as being dead”, therefore, it may be more helpful to think of it as a period of life that is barely worth living, or as bad as prolonged unconsciousness. We use the phrase “as bad as being dead” since this is the standard phrase used in HALY valuation exercises and we want to align the wellbeing QALY scale to the HALY scale.

^5^
However, parallel arguments can of course be made that there may be “spillovers” in wellbeing effects from one year to another and that the pattern of wellbeing over a lifetime matters.

^6^
Appendix S1D shows a comparable figure for a different set of parameter assumptions tailored to a UK context.

^7^
LifeSim (https://equipol.org/research/projects/lifesim) simulates a wide range of life outcomes, including skills, education, employment, earnings, health‐related behaviors, mental and physical illness, taxes, benefits, and various adverse life events. It generates a yearly dataset of the life outcomes over the lives of the simulated cohort members, from birth up to their death.
