# Supplementary material for: Quality adjusted life years based on health and consumption: A summary wellbeing measure for cross‐sectoral economic evaluation
Source: Health Econ. 2020 Oct 22;30(1):70–85. doi: 10.1002/hec.4177 (PMC7756450; doi:10.1002/hec.4177)
Supplement: Supplementary file 1 — Supplementary Material [file HEC-30-70-s001.pdf]

S1: Supplementary Materials for “Quality Adjusted Life Years Based on Health and Consumption: A General Wellbeing Measure for Cross-Sectoral Economic Evaluation”

List of Appendices (S1)

A: Wellbeing QALY and the ‘Equivalence Approach’ 2
B: Allowing for Interactions Between Consumption and Health 5
C: Sensitivity Analysis Around Different Normative Parameter Values 8
C.1. Sensitivity to the Elasticity of the Marginal Value of Consumption 8
C.2. Sensitivity to the Minimal Consumption Parameter 9
C.3. Sensitivity to the Standard Consumption Parameter 10
D: Illustration of Value of Consumption Function for a UK setting 12
E: QALY vs. Monetary Valuation of Consumption Gains 14
F: Other Extensions 16
F.1. Measuring Social Progress 16
F.2. Measuring Social Inequality in Lifetime Wellbeing 16

List of Tables

Table D1: Wellbeing QALYs vs. unweighted monetary valuation using illustrative UK parameters .. 13

List of Figures

Figure B1: Wellbeing as a function of income and health with different values of “alpha” 7
Figure C1: Wellbeing under alternative assumptions about the ‘eta’ parameter 8
Figure C2: Wellbeing under alternative assumptions about minimal consumption 9
Figure C3: Wellbeing under alternative assumptions about standard consumption 11
Figure D1: Value of consumption in full health 12

Appendix A. Wellbeing QALY and the 'Equivalence Approach'

In this appendix we demonstrate that the wellbeing QALY measure is consistent with the life-metric utility defined by Canning (2013), but also involves imposing specific assumptions about the functional form of the individual utility function and reference levels for standard health and income. Construction of life-metric utility and hence our wellbeing QALY follows what is known in the welfare economics literature as the "equivalence approach" to constructing wellbeing measures (Deaton & Muellbauer, 1980; Fleurbaey & Blanchet, 2013; Fleurbaey & Maniquet, 2011). This is based on the idea that bundles of income, health and other attributes that individuals derive utility (wellbeing) from can be considered equally good as long as they are on the same indifference curve.

Our aim is exclusively normative: we aim to value bundles of consumption, health and lifespan using our wellbeing function, not also to predict individual behaviour using the same wellbeing function. In particular, we do not need to assume that individuals always make "optimal" long-term plans as if they were fully informed rational self-interested maximisers of total wellbeing over the lifetime with rational expectations and infallible computational abilities. We are therefore not constrained by the potential behavioural paradoxes that might emerge if one were to make that behavioural assumption.

Following Canning (2013), we assume a world with no uncertainty and three types of goods – traded goods, health and lifespan. An individual's lifetime profile of different traded goods consumed in different time periods is represented by the vector, $\mathbf{x}$ (with a corresponding vector of prices $\mathbf{p}$), their profile of different health quality attributes over time is represented by the vector, $\mathbf{H}$ (with an upper bound for each type j of health attribute $\mathbf{H_j} \leq \mathbf{H_{max_j}}$), and their lifespan in years is represented by the scalar, l. Individuals are also endowed with an exogenous flow of period-specific income over their lifetime, represented by the vector, $\mathbf{y}$. Hence, an individual can be described by her allocation of the bundle $(\mathbf{x}, \mathbf{H}, l)$ and her endowment of income. The expression $\mathbf{xp} \leq \mathbf{y}$ defines the set of individual budget constraints in each period i.e. it says that the individual cannot afford to consume more traded goods in a given period than their income in that period allows. The use of period-specific budget constraints allows us to assume that the period-specific flows of income and health quality are exogenously given and exogenously malleable, and our only concern is to value different lifetime bundles of these goods from a normative perspective rather than to predict long-term savings, consumption and health behaviours.

Canning (2013) makes a set of standard assumptions about individual preferences[1], most of which are respected by the function that we use as part of our wellbeing QALY framework to represent the individual value of health and consumption in equations (3) and (4) in the main manuscript text. One key difference however is that Canning assumes utility must always be positive (better than being dead), whereas like the standard HALY approach we allow the existence of states worse than being dead. States worse than being dead raise possible behavioural puzzles for rational choice theorists – for example, the question of why people in states worse than dead do not always kill themselves. However, as our aim is to value wellbeing rather than to predict behaviour, this issue does not constrain us.

In Canning's framework, when the individual consumes a bundle of traded goods **x,** her indirect utility from income, health and life-span can be represented by $v(\mathbf{y}, \mathbf{H}, l) = \max_{x}\{U(\mathbf{x}, \mathbf{H}, l) | (\mathbf{x}, \mathbf{H}, l) \in F, \mathbf{xp} \leq \mathbf{y}\}$. This means that, given prices, the individual indirect utility can be defined as a function of income, health attributes and lifespan. For brevity, we have omitted the price vector **p** from the list of arguments of v().

Consistent with the equivalence approach, Canning (2013) derives his life-metric utility l^* using a hypothetical trade-off between the individual's actual life with their actual lifespan, income and health profile and a hypothetical life with assumed reference levels of "good" of income and full health. More specifically, l^* is defined as the hypothetical number of life years in full (reference-level) health and consuming the reference level of income, that the individual would find equally good as their actual bundle of health and income over their actual life span. Life-metric utility l^* is implicitly defined by the condition $v(\mathbf{c_{ref}}, \mathbf{H_{ref}}, l^*) = v(\mathbf{c}, \mathbf{H}, l)$, which we further refer to as the 'equivalence condition'.

$$v(\mathbf{c_{ref}}, \mathbf{H_{ref}}, l^*) = v(\mathbf{c}, \mathbf{H}, l) \qquad \text{(A1)}$$

This condition states that the utility derived from consuming the reference bundle with a life span of l^* is the same as the utility derived from consuming the actual bundle of income and health for the actual life span.

Canning proves the uniqueness and existence of l^* for the general case that satisfies his assumptions. In our specific case with the specific utility function defined by equations (3) and (4) in the main paper text, it is straightforward to come up with a similar argument and derive l^* from the equivalence condition. We show that, together with our assumptions laid out in the main text of the paper, this l^* represents our wellbeing QALY measure.

[1] They are represented on the bounded set of all the possible bundles *F* by a continuous utility function U increasing in each argument, and strictly increasing in life span *l*; this latter assumption is however rules out the possibility of some health states being valued worse than being dead; and also similarly rules out states with consumption so low which would make 'life not worth living'.

More specifically, we assume that:

- the multidimensional health attributes $\mathbf{H}_\mathrm{t}$ in time period t – can be summarised in the standard way by $h_t(\mathbf{H}_\mathrm{t})$, where h_t is scalar measured on the standard scale of HALY;[2]
- the reference level consumption is the standard consumption parameter c_{std} in each year of life, and reference health is full health as represented by the maximum health quality of 1 in each year of life, i.e. $c_{ref} = c_{std}$ and $h_{ref} = h_{max} = 1$ in each time period;
- the period specific individual utility function is defined by equations (3) and (4) (in main text) with health quality and utility of consumption is additively separable over time.

With these assumptions, the equivalence condition in A1 can be represented by:

$$\sum_{t=1}^{l^*}\{1 + A - B \times c_{std}^{1-\eta} - 1\} = \sum_{t=1}^{l}\{h_t + A - B \times c_t^{1-\eta} - 1\} \qquad \text{(A2)}$$

The '$1 - 1$' cancels out; then after substituting the full expressions in terms of c_{std} and c_{min} for the normalisation constants A, B on the left-hand-side of the condition (A2), it becomes clear that $A - B \times c_{std}^{1-\eta} = 1$ and it reduces to the expression defining lifetime good life years in terms of wellbeing QALYs:[3]

$$l^* = \sum_{t=1}^{l}\{h_t + A - B \times c_t^{1-\eta} - 1\} \qquad \text{(A3)}$$

We have demonstrated that the wellbeing-QALY measure can also be shown to arise from a hypothetical trade-off between the individual's actual levels of lifetime income and health, and assumed reference levels of "good" of income and full health, which is based on the equivalence approach of wellbeing valuation. Therefore, the wellbeing QALY can also be interpreted as a measure of equivalent life, and it is consistent with the framework laid out by Canning (2013) but with more specific assumptions about the functional form and the reference levels for full health and consumption.

[2] Bounded above at 1, where 1 represents full health and 0 represents a state as bad as being dead.

[3] Recall that $A = c_{min}^{(1-\eta)} / (c_{min}^{(1-\eta)} - c_{std}^{(1-\eta)})$ and $B = 1 / (c_{min}^{(1-\eta)} - c_{std}^{(1-\eta)})$.

Appendix B. Allowing for Interactions Between Consumption and Health

The simple additive wellbeing function assumes the marginal benefit of consumption does not depend on ill health (Bleichrodt & Quiggin, 1999; Hammitt, 2013; Smith & Keeney, 2005). An alternative view might be that the marginal benefit of consumption increases with ill health. For example, someone unable to walk might gain considerable benefit from consuming mobility equipment and a variety of transport, communication and personal care services – at least some of which might not be picked up in standard measures of health benefit. Yet another view might be that the marginal benefit of consumption decreases with ill health. For example, additional consumption of material goods and services may bring limited benefit to someone who is severely depressed and no longer able to enjoy material consumption.

To allow for these possibilities, a more general wellbeing function would have the following form, based on a weighted average of additive and multiplicative functional forms:

$$w_{i,t} = \alpha + \alpha\, h_{i,t}\, u(c_{i,t}) + (1-\alpha)\left(h_{i,t} + u(c_{i,t})\right) - 1 \quad \text{(B1)}$$

- where $u(c_{i,t})$ is the wellbeing of consumption in full health;
- α ('alpha') is a consumption-health interaction parameter, bounded above by 1 to ensure that the marginal utility of consumption is always positive so long as health is positive.

When $\alpha = 0$, this reduces to the additive form in equation (3) (in the main text). When $\alpha > 0$ the marginal benefit of consumption decreases with ill health, so that health and consumption function like economic complements. When $\alpha < 0$ the marginal benefit of consumption increases with ill health, so that health and consumption function like economic substitutes. This form is uniquely determined by the assumptions that: (i) the gambles people would accept over consumption levels are independent of quality of health state; (ii) the gambles people would accept over health states are independent of consumption level; and the boundary conditions (iii) if $u(c_{i,t}) = h_{i,t} = 1$ then $w_{i,t} = 1$; (iv) if $u(c_{i,t}) = 1$, $h_{i,t} = 0$, then $w_{i,t} = 0$; and (v) if $u(c_{i,t}) = 0$, $h_{i,t} = 1$, then $w_{i,t} = 0$. There is not much empirical evidence about this issue (Evans & Viscusi, 1991; Rey & Rochet, 2004), but one study suggested a positive value (Viscusi & Evans, 1990) whereas a more recent study supports a negative value of alpha of around -1 (Tengstam, 2014).

In practice, we would therefore propose using a base case assumption of alpha $\alpha = 0$, for convenience and simplicity, and then sensitivity analysis around alternative plausible values such as $\alpha = 0.5$ (in the middle of the possible range up to 1) and $\alpha = -1$.

Figure B1 shows how wellbeing changes with different levels of health under different assumptions about alpha, returning to our base case assumption that minimal consumption is subsistence consumption.[4] The lowest health quality score is -0.281, reflecting the lowest score from the latest EQ-5D-5L health value set for England (Devlin, Shah, Feng, Mulhern, & van Hout, 2016).

[4] A web-based application for drawing further graphs of this kind based on different parameter values is available here https://miqdadasaria.shinyapps.io/wellbeing_adjusted_life_years/

Figure B1: Wellbeing as a function of income and health with different values of "alpha"

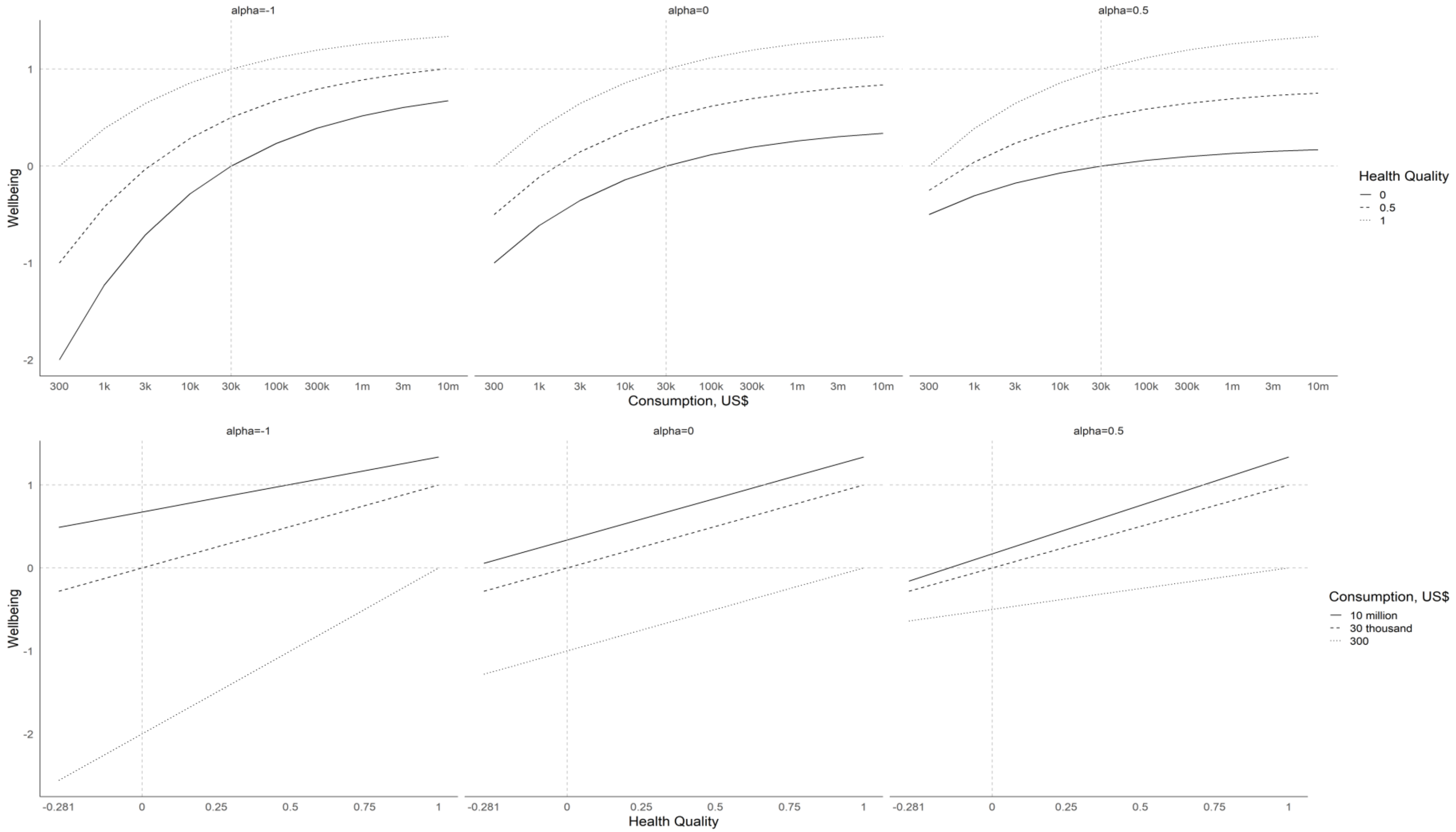

Note: *Consumption is shown on a log scale. The wellbeing metric is based on the specification that allows for interaction between health and consumption. The assumptions for the fixed parameters are as follows: minimal consumption (for a life worth living)* c_{min}*=$300, standard consumption (for a good life)* c_{std}*=$30,000, and elasticity of the marginal value of consumption* η *= 1.26.*

Appendix C. Sensitivity Analysis Around Different Normative Parameter Values

C.1. Sensitivity to the Elasticity of the Marginal Value of Consumption

Figure C1 shows how the wellbeing function changes with different assumed values for the "eta" parameter. When $\eta = 0$, the wellbeing function is linear and exhibits constant marginal value of consumption. For $\eta > 0$, the function exhibits diminishing marginal value. The higher the "eta", the more concave the function is which here means that more wellbeing value is attached to consumption when it is scarce (i.e. the case of a poor person) relative to when it is abundant (i.e. the case of a rich person). Notice that for $\eta \leq 1$, the value of consumption is always increasing, and has no upper bound, even though the increase may get infinitesimally small as consumption approaches infinity. For $\eta > 1$, the value of consumption has an upper bound. In Figure C1 it can be seen that the marginal value of consumption for the curve with $\eta = 2$ approaches zero for consumption levels above the assumed standard consumption of $30,000.

Figure C1: Wellbeing under alternative assumptions about the 'eta' parameter

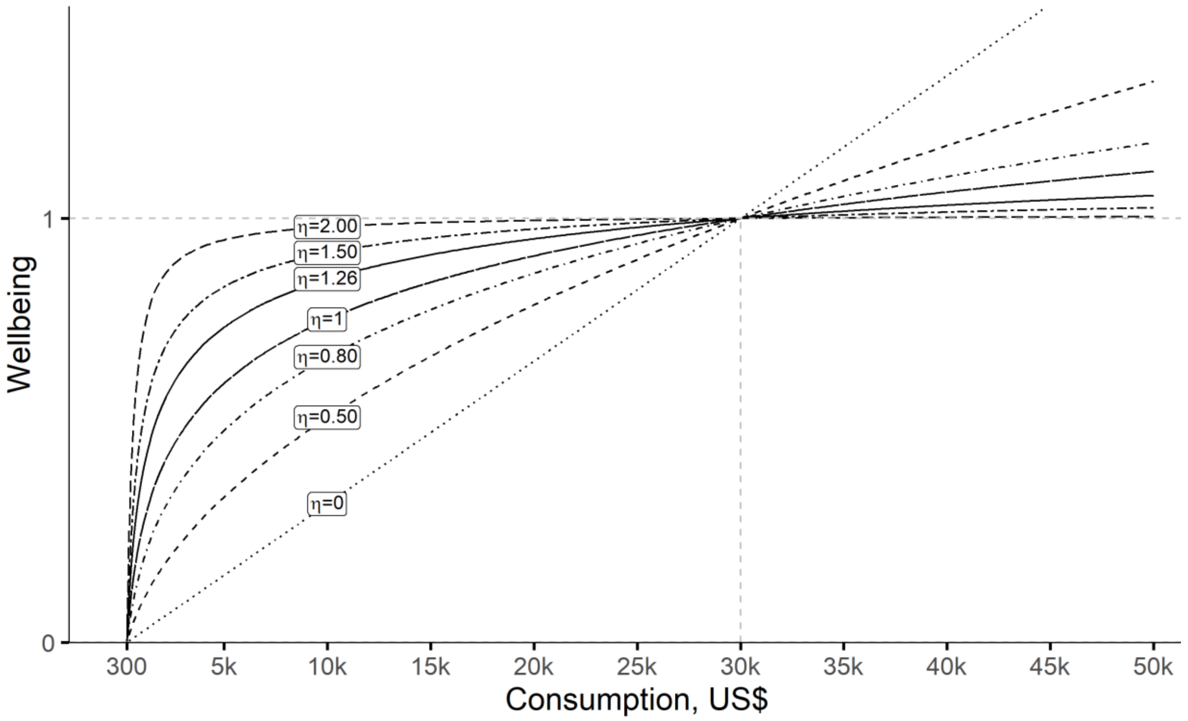

Note: *The wellbeing metric is based on the additive specification described in section 2.3. The assumed elasticity of the marginal value of consumption (eta parameter- η) varies by each curve and is given in the box on the corresponding curve. The other parameters are fixed for all of the curves as follows: minimal consumption (for a life barely worth living) c_{min}=$10,000, standard consumption (for a good living standard) c_{std}=$30,000.*

C.2. Sensitivity to the Minimal Consumption Parameter

We illustrate the implications of increasing c_{min} to a level well above the subsistence level of the global absolute poverty line. An alternative assumption would be to raise c_{min} to a high-income country relative poverty line. For example, this could be set equal to a minimal level of consumption considered acceptable for the poorest citizen in a high-income country, such as the consumption of an unemployed adult with no private wealth who relies entirely on state benefits and services. To illustrate this alternative assumption, the dashed curve in Figure C2 below explores the value of c_{min} = \$10,000 per year. This assumption implies that human life is barely worth living at the poverty line in a high-income country.

Figure C2 shows that increasing minimal consumption from \$300 to \$10,000 has shifted the wellbeing curve, in such a way that the year of life is of a lesser quality for those with annual income less than \$30,000 (the assumed standard consumption) and of a greater quality for those with annual consumption above \$30,000. Also, those with consumption less than \$10,000 now have a negative value for their wellbeing, which is consistent with the assumption that below this level of consumption life is no longer worth living even in full health.

Figure C2: Wellbeing under alternative assumptions about minimal consumption.

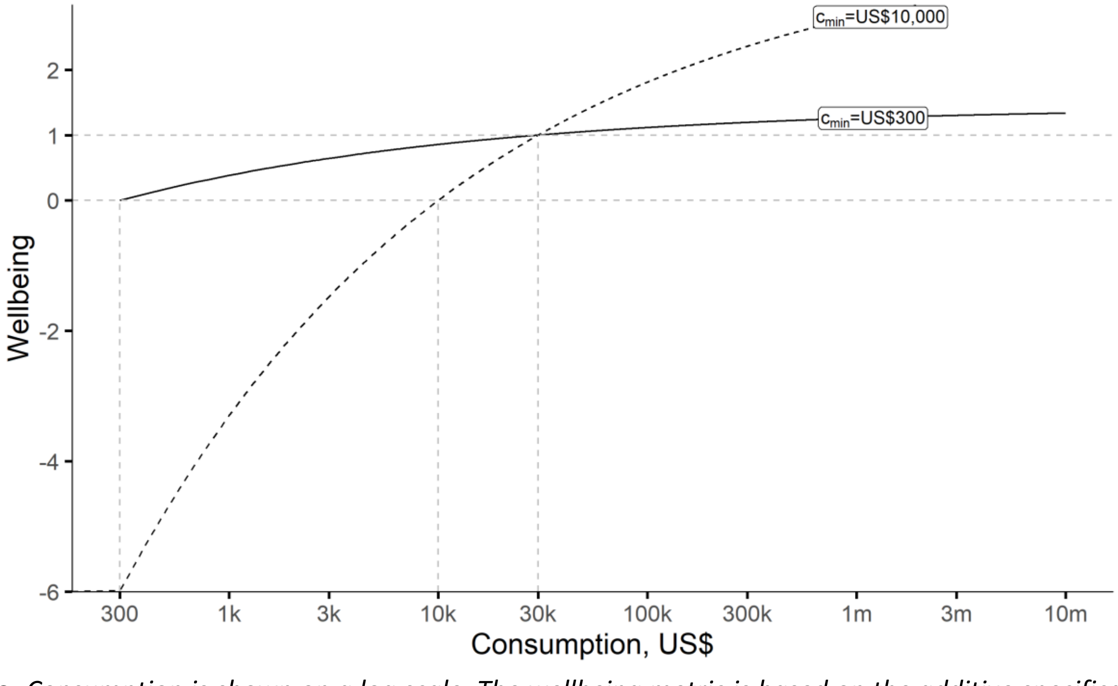

Note: *Consumption is shown on a log scale. The wellbeing metric is based on the additive specification described in section 2.3, and depicted for a person in full health. Minimal consumption (for a life barely worth living) varies across the two curves, i.e.* c_{min}*=\$10,000 for the dashed curve,* c_{min}*=\$300 for the solid curve. The rest of the parameters are fixed for both curves with standard consumption (for a good living standard)* c_{std}*=\$30,000, and elasticity of the marginal value of consumption* η *= 1.26.*

If the wellbeing function $w(.)$ has the additive specification described in section 2.3., its first derivative with respect to the minimal consumption parameter is $w'(c_{min}) = \frac{(1-\eta)\left(c^{1-\eta}-c_{std}^{1-\eta}\right)}{c_{min}^{\eta}\left(c_{min}^{1-\eta}-c_{std}^{1-\eta}\right)^2}$. The sign of this term reveals how the wellbeing function changes given the value for the minimal consumption parameter.

When $c = c_{std}$, then $w'(c_{min}) = 0$ - the value of the wellbeing function is fixed at 1 and independent from the minimal consumption parameter.

At other consumption levels $c \neq c_{std}$, it can be shown that $\begin{cases} w'(c_{min}) < 0 \text{ for } c < c_{std} \\ w'(c_{min}) > 0 \text{ for } c > c_{std} \end{cases}$.

This means that the wellbeing function is decreasing in the c_{min} parameter, in the range where the consumption level is below the standard consumption; and increasing in the c_{min} parameter whenever consumption is above the standard consumption (as can be seen in Figure C2).

Notice that this holds for all $\eta > 0$, because:

- when $0 \leq \eta < 1$, then the term $1 - \eta > 0$, and the term $c^{1-\eta} - c_{std}^{1-\eta} > 0$ for $c > c_{std}$ and $c^{1-\eta} - c_{std}^{1-\eta} < 0$ for $c < c_{std}$. This happens as the function $f(x) = x^{1-\eta}$ is increasing in x when $\eta < 1$;

- when $\eta > 1$, then the term $1 - \eta < 0$, and the term $c^{1-\eta} - c_{std}^{1-\eta} < 0$ for $c > c_{std}$ and $c^{1-\eta} - c_{std}^{1-\eta} > 0$ for $c < c_{std}$, as the function $f(x) = x^{1-\eta}$ is decreasing in x when $\eta > 1$.

C.3. Sensitivity to the Standard Consumption Parameter

Figure C3 shows the wellbeing function for different parameters of standard consumption. As we decrease the value of c_{std} from \$30,000 to \$20,000, the wellbeing function becomes steeper and returns higher values for the same level of consumption; as we increase the value of c_{std} from \$30,000 to \$100,000 the wellbeing function becomes less steep and returns lower wellbeing values for the same level of consumption.

To formally show how the wellbeing function changes for different values of c_{std}, similar to the previous section, we analyse the first derivative of $w(.)$ with respect to c_{std}:
$w'(c_{std}) = \frac{(1-\eta)\left(c_{min}^{1-\eta}-c^{1-\eta}\right)}{c_{std}^{\eta}\left(c_{min}^{1-\eta}-c_{std}^{1-\eta}\right)^2}$.

When $c = c_{min}$, then $w'(c_{std}) = 0$ - the wellbeing is fixed at zero, and is independent from the parameter c_{std}.

Furthermore, it can be shown that $\begin{cases} w'(c_{std}) > 0 \text{ for } c < c_{min} \\ w'(c_{std}) < 0 \text{ for } c > c_{min} \end{cases}$, which means that the wellbeing function is decreasing in c_{std} in the range where consumption exceeds the minimal consumption (as can be seen in Figure C3); and increasing in c_{std} whenever consumption falls below the minimal consumption.

Figure C3: Wellbeing under alternative assumptions about standard consumption

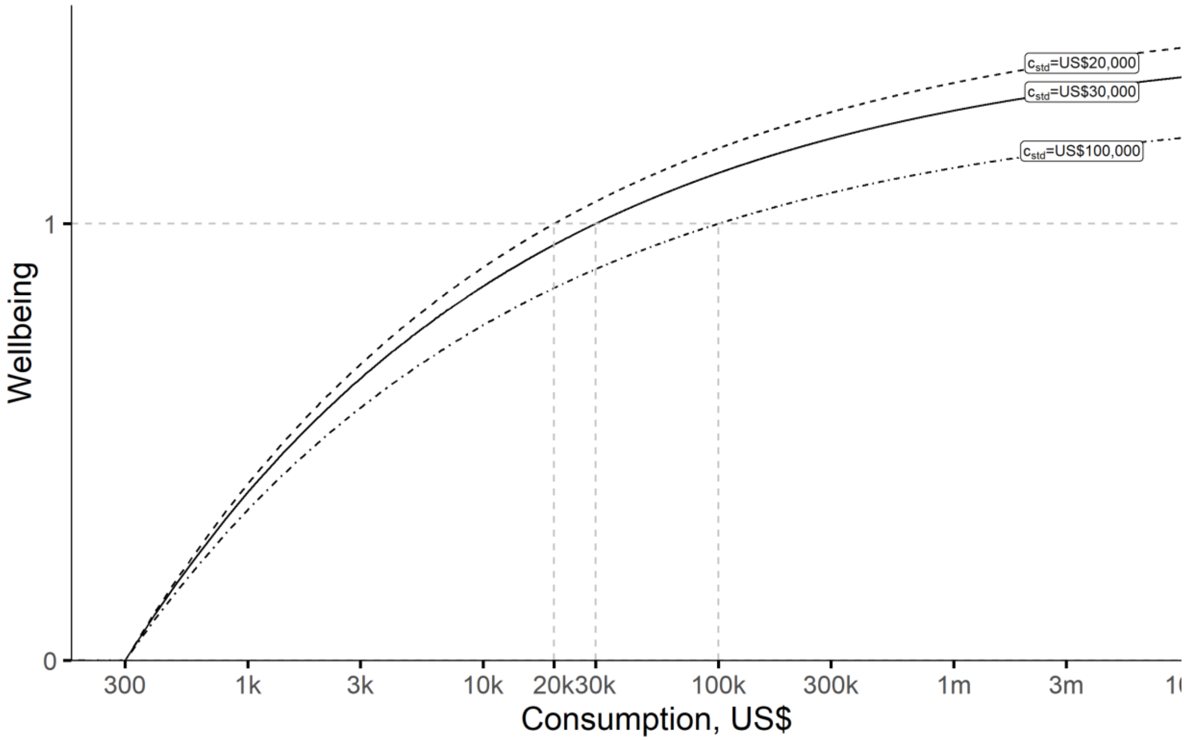

Note: *Consumption is shown on a log scale. The wellbeing metric is based on the additive specification described in section 2.3, for a person in full health. Standard consumption (for a good standard of living) varies across the three curves, i.e.* c_{std}*=$20,000 for the top (dashed) curve,* c_{std}*=$30,000 for the middle (solid) curve, and* c_{std}*=$100,000 for the bottom (dash-dotted) curve. The rest of the parameters are fixed for all curves with minimal consumption (for a life barely worth living)* c_{min}*=$300, and elasticity of the marginal value of consumption* η *= 1.26.*

Appendix D. Illustration of Value of Consumption Function for a UK Setting

Figure D1: Value of consumption in full health

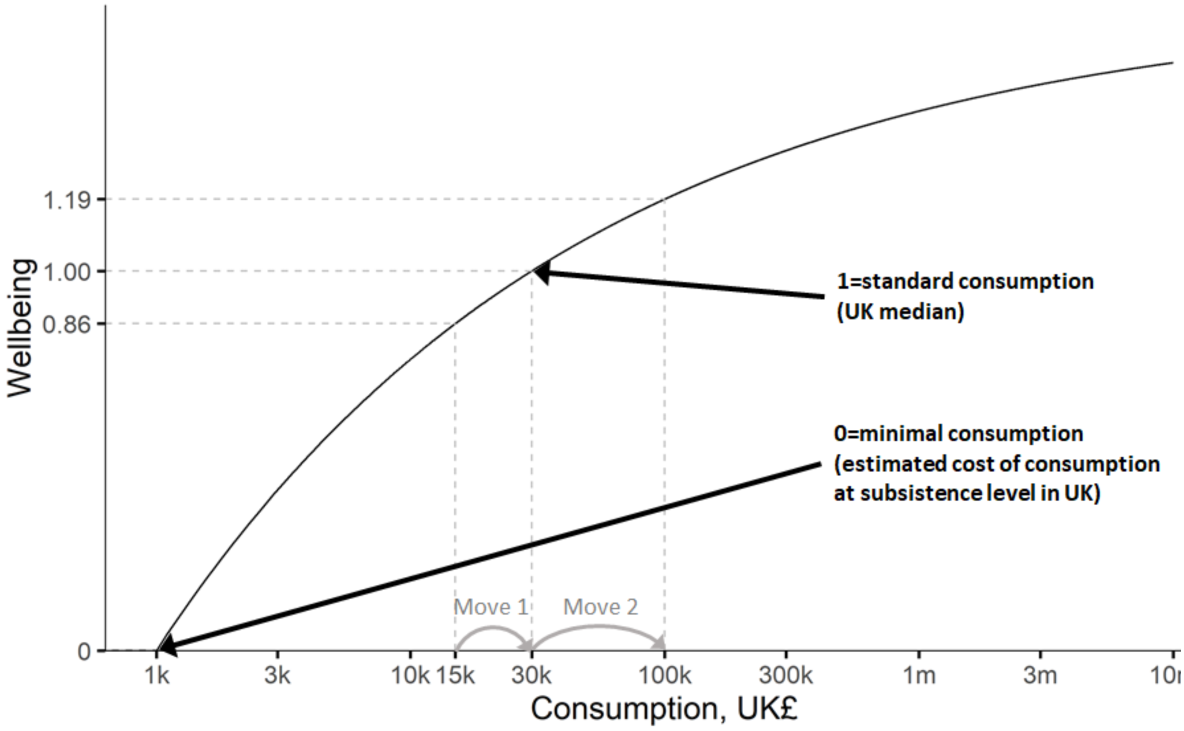

Note: *Consumption shown on a log scale. Wellbeing is depicted for a person in full health, assuming the following parameters: minimal consumption (for a life barely worth living)* c_{min}*=£1,000, standard consumption (for a good living standard or "prosperity")* c_{std}*=£30,000 and elasticity of the marginal value of consumption* η *= 1.26. We also mark the "poverty consumption" at* c_{pov} *= £15,000 and "affluence line" consumption at* c_{aff} *= £100,000. Move 1 depicts an improvement in living standard from poverty consumption to standard consumption ("poverty-to-prosperity"), and move 2 an improvement from standard consumption to affluent consumption ("prosperity-to-affluence").*

Table D1: Wellbeing QALYs vs. unweighted monetary valuation using illustrative UK parameters

Concept	*Poverty-to-prosperity*	*Prosperity-to-affluence*
**Definition**	*One person's annual consumption improved from £15,000 to £30,000*	*One person's annual consumption improved from £30,000 to £100,000*
**Gain, unweighted UK£**	15,000	70,000
Relative valuation	0.21 (prosperity-to-affluence is 476% more important than poverty-to-prosperity)	
**Gain, wellbeing QALYs**	0.14	0.19
Relative valuation	0.74 (prosperity-to-affluence is 35% more important than poverty-to-prosperity)	

Note: *The "relative valuation" represents the gains from "poverty-to-prosperity" divided by the gains from "prosperity-to-affluence", as defined above. The above wellbeing calculations are for a person in full health, assuming the following parameters: minimal consumption (for a life barely worth living)* c_{min}*=£1000, standard consumption (for a good living standard or "prosperity")* c_{std}*=£30,000 and elasticity of the marginal value of consumption* $\eta = 1.26$*. The two ratios of gains (for QALYs and unweighted UK£) will remain the same independently from the parameters* c_{min}, c_{std} *and the QALY relative valuation will always be larger than the monetary one, so long as there are diminishing returns to consumption i.e.* η *is greater than 0 (see discussion and proof in Appendix S1E).*

Appendix E. QALY vs. Monetary Valuation of Consumption Gains

We generalise the illustrative examples of QALY versus monetary valuation of consumption gains that are presented in section 3.2. of the main paper, and Appendix S1D.

The example in the main paper used normative parameter values tailored to a global context and the absolute global poverty line, whereas the example in Appendix S1D was tailored to a UK context and the (substantially higher) UK relative poverty line. The global example compared (1) increasing the annual consumption of a hypothetical person from absolute poverty ($600) to a prosperous standard of living ($30,000) ("poverty-to-prosperity") with (2) increasing the consumption of another hypothetical person from a good to an affluent standard of living $30,000 to $100,000 ("prosperity-to-affluence"). It showed that "poverty-to-prosperity" is about 7 times more valuable than "prosperity-to-affluence" when using the wellbeing QALY approach, but about 2.5 times less valuable under monetary valuation. The example in Appendix S1D used normative parameter values tailored to a UK context, including use of a UK poverty line of £15,000 annual consumption that is substantially higher than the absolute global poverty line of $600 annual consumption. The relative valuations were substantially different. However, this was entirely due to differences in the thresholds used to define "poverty" and "affluence" in global versus high-income country settings, rather than differences in the normative parameter values used to define minimal consumption and standard consumption.

We draw general conclusions about how the QALY relative valuation of a "poverty-to-prosperity" gain relative to a "prosperity-to-affluence" gain will depend on the chosen normative parameters, for any given specification of the "poverty" and "affluence" thresholds. Specifically, we show that:

1) the QALY relative valuation does not depend on standard consumption or minimal consumption;
2) the QALY relative valuation will always be higher than the monetary relative valuation so long as the elasticity of the marginal value of consumption is above zero.

In other words, so long as there are diminishing returns to consumption, QALY valuation will always attach relatively more importance to "poverty-to-prosperity" than monetary valuation, even in cases such as the UK example where "poverty-to-prosperity" is valued less highly than "prosperity-to-affluence".

Let's denote four consumption levels expressed in monetary terms as $x_0,\ x_1,\ z_0,\ z_1$, such that $0 \le x_0 < x_1 \le z_0 < z_1$. This is consistent with our example in section 3.2., where $x_0 = \$600,\ x_1 = z_0 = \$30{,}000$, and z_1=$100,000.

Let's define the difference $x_1 - x_0$ as "move 1" ("poverty-to-prosperity") and $z_1 - z_0$ as "move 2" ("prosperity-to-affluence").

The monetary relative valuation of move 1 relative to move 2 is $\frac{x_1 - x_0}{z_1 - z_0}$; the QALY relative valuation is $\frac{w(x_1) - w(x_0)}{w(z_1) - w(z_0)}$. Assuming $w(.)$ is the additive specification described in section 3.2. of the main article, the QALY relative valuation can be simplified as follows: $\frac{w(x_1) - w(x_0)}{w(z_1) - w(z_0)} = \frac{x_1^{1-\eta} - x_0^{1-\eta}}{z_1^{1-\eta} - z_0^{1-\eta}}$ which shows that this expression is independent from the parameters c_{min} and c_{std}. If $\eta = 1$, the function $w(.)$ is of logarithmic form, such that the ratio is $\frac{ln(x_1) - ln(x_0)}{ln(z_1) - ln(z_0)}$; in this case the ratio is also independent from the parameters c_{min} and c_{std}.

If $\eta \neq 1$, the ratio depends on the parameter η, and below we show that for any $\eta \geq 0$, it will hold that $\frac{x_1^{1-\eta} - x_0^{1-\eta}}{z_1^{1-\eta} - z_0^{1-\eta}} \geq \frac{x_1 - x_0}{z_1 - z_0}$, i.e. the corresponding QALY relative valuation is always larger or equal in value to the relative valuation in monetary terms.

For $\eta = 0$, this result will hold, as the QALY relative valuation becomes $\frac{x_1 - x_0}{z_1 - z_0}$. In other words, the wellbeing function is linear in consumption implying no diminishing marginal value of consumption, so the analysis is identical to the monetary approach.

To see why the result holds for $\eta > 0$, note that for a function $f(y) = y^{1-\eta}$, the first derivative is $f'(y) = (1-\eta)y^{-\eta}$ and the second derivative $f''(y) = -\eta(1-\eta)y^{-\eta-1}$, therefore:

- for $0 < \eta < 1$, it holds that $f'(y) > 0$ and $f''(y) < 0$ which means that the function $f(y)$ is increasing and concave (increasing at a diminishing rate), so when $0 \leq x_0 < x_1 \leq z_0 < z_1$ it holds that $\frac{f(x_1) - f(x_0)}{f(z_1) - f(z_0)} > \frac{x_1 - x_0}{z_1 - z_0}$, i.e. $\frac{x_1^{1-\eta} - x_0^{1-\eta}}{z_1^{1-\eta} - z_0^{1-\eta}} > \frac{x_1 - x_0}{z_1 - z_0}$;
- for $\eta = 1$, the function $f(y)$ is logarithmic, so $f'(y) > 0$ and $f''(y) < 0$, and the same result holds;
- for $\eta > 1$, it holds that $f'(y) < 0$ and $f''(y) > 0$ – the function is decreasing and convex (decreasing at a diminishing rate), so in this case again when $0 \leq x_0 < x_1 \leq z_0 < z_1$ it holds that $\frac{x_1^{1-\eta} - x_0^{1-\eta}}{z_1^{1-\eta} - z_0^{1-\eta}} > \frac{x_1 - x_0}{z_1 - z_0}$.

Therefore, for any $\eta > 0$, when $0 \leq x_0 < x_1 \leq z_0 < z_1$ it is true that $\frac{x_1^{1-\eta} - x_0^{1-\eta}}{z_1^{1-\eta} - z_0^{1-\eta}} > \frac{x_1 - x_0}{z_1 - z_0}$.

Appendix F. Other Extensions

F.1. Measuring Social Progress

It is also possible to use our wellbeing metric to measure social progress. The simplest way of doing this is to calculate average lifetime wellbeing. Like consumption, average lifetime wellbeing can measured on a ratio scale. Unlike an interval scale (e.g. degrees Fahrenheit), a ratio scale has an absolute zero and so it makes sense to calculate ratios and percentage differences. This is useful for measuring social progress, since it allows the calculation of percentage changes (growth or decline) in a society's average lifetime wellbeing over time, and percentage differences in average lifetime wellbeing between different societies.

Not all measures of social progress allow this. For example, the concept of "full national income" augments standard measures of change in national income by adding in the monetary value of changes in population health over time (Jamison et al., 2013).[5] Unfortunately, however, this only allows one to calculate changes in full national income over time, not baseline levels of full national income. So unlike wellbeing QALYs based on consumption and health, the concept of "full national income" does not allow comparison of percentage changes over time or percentage differences between societies.

F.2. Measuring Social Inequality in Lifetime Wellbeing

Our approach already embodies one important type of concern for social inequality. The wellbeing QALY based on consumption and health embodies an assumption of diminishing marginal value of consumption. It thus embodies the same form of concern for inequality in consumption as classical utilitarianism. The utilitarian case for redistribution is that a dollar of consumption is worth less to a rich person than a poor person – hence, other things equal, taking a dollar from a rich person and giving it to a poor person will tend to increase sum total wellbeing.

However, policymakers may have additional concerns for social inequality in lifetime wellbeing, as well as inequality in current consumption. Our wellbeing QALY metric is well suited to analysing such concerns, for three reasons. First conducting separate analyses of inequality in different components of wellbeing – e.g. inequality in consumption and inequality in health – may be misleading, insofar as different components of wellbeing can compensate for one another (Adler, 2012; Fleurbaey & Schokkaert, 2009). Second, a ratio scale measure of individual lifetime wellbeing allows the use of standard indices of relative inequality based on percentage differences between individuals. Third, the wellbeing QALY is well suited to analysing trade-offs between "efficiency" in terms of average wellbeing versus "equity" in terms of reducing inequality in the social distribution of lifetime wellbeing. The wellbeing QALY metric allows the use of standard social welfare functions to analyse equity-efficiency

[5] See, in particular, supplementary appendix 3: http://globalhealth2035.org/report/supplementary-web-appendices

trade-offs of this kind. Standard social welfare functions can be expressed in the following abbreviated or reduced form (Adler, 2012):

$$W = \bar{w} * (1 - I(\boldsymbol{w}, \varepsilon)) \quad \text{(F1)}$$

- where W is social welfare;
- $\boldsymbol{w}$ is a vector of the individual lifetime wellbeing of all individuals or groups in society;
- $\bar{w}$ is mean individual lifetime wellbeing across the whole population;
- I(.) is an inequality index scaled from 0 to 1 (where 0 is full equality and 1 full inequality);
- ε is an inequality aversion parameter.

One plausible functional form for the inequality index is the Atkinson function (Adler, 2012) (see Chapter 5). In the Atkinson function, $\varepsilon = 0$ represents zero aversion to inequality in lifetime wellbeing in which case I = 0. Higher values of ε imply greater weight to the worse off i.e. those with lower lifetime wellbeing. Finally, an infinite value of ε implies exclusive priority to the worst-off individual or group – i.e. a "maximin" principle. Once ε has been specified, it is then possible to compare populations and policies in terms of overall social wellbeing, and to analyse trade-offs between changes in average lifetime wellbeing, $\bar{w}$, and changes in inequality, I.

An extension of this approach is to adjust the vector of lifetime wellbeing as appropriate to focus on "unfair" determinants of individual wellbeing (e.g. parental class or race) and to set aside "fair" determinants (e.g. personal responsibility) and determinants that are neither "fair" nor "unfair" (e.g. misfortunes considered a matter of personal tragedy rather than social injustice) (Adler, 2012; Asada, Hurley, Norheim, & Johri, 2015; Ferriera & Peragine, 2016; Fleurbaey & Schokkaert, 2009, 2011) (see Chapter 8 of Adler).

Analysing inequality in lifetime wellbeing QALYs does not preclude performing additional forms of distributional analysis. Decision makers may still want to have information about dimension-specific inequality in consumption, for example if they have non-utilitarian concerns about inequality in consumption. And they may want information about dimension-specific inequality in health if they have special concerns for inequality in health. For example, in 1997, the then UK Secretary of State for Health Frank Dobson said that "Health inequality is the worst inequality of all. There is no more serious inequality than knowing that you'll die sooner because you're badly off" (http://www.lgcplus.com/govt-takes-action-to-reduce-health-inequalities/1494985.article). Our framework complements dimension-specific analyses of this kind, by analysing interactions between consumption, health and wellbeing and placing the analysis within a more general framework.

Supplementary Materials References

Adler, M. D. (2012). *Well-being and fair distribution: beyond cost-benefit analysis*. New York, US: Oxford University Press.

Asada, Y., Hurley, J., Norheim, O., & Johri, M. (2015). Unexplained health inequality - is it unfair? *International Journal for Equity in Health, 14*(1), 11.

Bleichrodt, H., & Quiggin, J. (1999). Life-cycle preferences over consumption and health: when is cost-effectiveness analysis equivalent to cost–benefit analysis? *Journal of Health Economics, 18*(6), 681-708.

Canning, D. (2013). Axiomatic foundations for cost-effectiveness analysis. *Health Economics, 22*(12), 1405-1416.

Deaton, A., & Muellbauer, J. (1980). *Economics and consumer behavior*. New York, US: Cambridge University Press.

Devlin, N., Shah, K., Feng, Y., Mulhern, B., & van Hout, B. (2016). Valuing Health-Related Quality of Life: An EQ-5D-5L Value Set for England.

Evans, W. N., & Viscusi, W. K. (1991). Estimation of state-dependent utility functions using survey data. *The Review of Economics and Statistics*, 94-104.

Ferriera, F. H. G., & Peragine, V. (2016). Individual Responsibility and Equality of Opportunity (Chapter 25). In M. D. Adler & M. Fleurbaey (Eds.), *The Oxford Handbook of Well-Being and Public Policy*: Oxford University Press.

Fleurbaey, M., & Blanchet, D. (2013). *Beyond GDP: Measuring welfare and assessing sustainability*. New York, US: Oxford University Press.

Fleurbaey, M., & Maniquet, F. (2011). *A theory of fairness and social welfare* (Vol. 48). New York, US: Cambridge University Press.

Fleurbaey, M., & Schokkaert, E. (2009). Unfair inequalities in health and health care. *Journal of Health Economics, 28*(1), 73-90. doi:http://dx.doi.org/10.1016/j.jhealeco.2008.07.016

Fleurbaey, M., & Schokkaert, E. (2011). Chapter Sixteen - Equity in Health and Health Care. In T. G. M. Mark V. Pauly & P. B. Pedro (Eds.), *Handbook of Health Economics* (Vol. Volume 2, pp. 1003-1092): Elsevier.

Hammitt, J. K. (2013). Admissible utility functions for health, longevity, and wealth: integrating monetary and life-year measures. *Journal of Risk and Uncertainty, 47*(3), 311-325.

Jamison, D. T., Summers, L. H., Alleyne, G., Arrow, K. J., Berkley, S., Binagwaho, A., . . . Frenk, J. (2013). Global health 2035: a world converging within a generation. *The Lancet, 382*(9908), 1898-1955.

Rey, B., & Rochet, J.-C. (2004). Health and wealth: How do they affect individual preferences? *The Geneva Papers on Risk and Insurance Theory, 29*(1), 43-54.

Smith, J. E., & Keeney, R. L. (2005). Your money or your life: A prescriptive model for health, safety, and consumption decisions. *Management Science, 51*(9), 1309-1325.

Tengstam, S. (2014). Disability and marginal utility of income: Evidence from hypothetical choices. *Health Economics, 23*(3), 268-282.

Viscusi, W. K., & Evans, W. N. (1990). Utility functions that depend on health status: estimates and economic implications. *The American Economic Review*, 353-374.
